# Do the ball-ended probe cause less damage than sharp explorers?—An ultrastructural analysis

**DOI:** 10.1186/s12903-016-0197-9

**Published:** 2016-03-22

**Authors:** Juliana Mattos-Silveira, Marina Monreal Oliveira, Ronilza Matos, Cacio Moura-Netto, Fausto Medeiros Mendes, Mariana Minatel Braga

**Affiliations:** Department of Pediatric Dentistry, Dental School, University of Sao Paulo, Av. Lineu Prestes, 2227, 05508-000 São Paulo, São Paulo Brazil; Dental School, Fundação Hermínio Ometto, Uniararas, Av. Dr. Maximiliano Baruto, 500, 13607-339 Araras, São Paulo Brazil; Dental School, Guarulhos University, Praça Tereza Cristina, 88, 07023-070 Guarulhos, São Paulo Brazil; Dental School, Cruzeiro do Sul University, Rua Galvão Bueno, 868, 01506-000 São Paulo Sao Paulo, Brazil

**Keywords:** Caries lesions, Primary tooth, Detection, Tactile examinations, Explorer, Ball-ended probe

## Abstract

**Background:**

No evidence about damage caused by ball-ended probes on tooth is available. No study compared probing defects caused by ball-ended probes with sharp explorers during tactile examinations of primary teeth. This exploratory study aimed to compare ultrastructural defects caused by ball-ended probes with sharp explorers during tactile examinations of primary teeth.

**Methods:**

Forty-nine primary extracted teeth were tactile examined as performed for caries activity assessment. Surfaces were randomly divided into groups based on probe type (ball-ended probe or sharp explorer). Two examiners probed different surfaces using the sharp explorer and the ball-ended probe. The order for examination was randomly determined. Images were captured using environmental scanning electron microscopy (ESEM) before and after probing. Two external examiners evaluated independently the ESEM images and scored them as: 0) no damage, 1) slight marks, 2) distinct marks, 3) marks with discontinuity, 4) enamel break-offs. Multilevel Poisson regression models were used to analyze associations between probing ultrastructural damage and surface type, baseline condition and probe type. Prevalence ratios (PR) were calculated with 95 % confidence interval (CI).

**Results:**

The most common defects observed on the dental surfaces were probing marks without discontinuity (scores 1 and 2). Ball-ended probes caused significantly less severe damage than sharp explorers (PR: 0.28; CI: 0.11–0.76, *p* = 0.01).

**Conclusion:**

Ball-ended probes cause less damage than sharp explorers when probing gently dental surfaces of primary teeth.

## Background

Visual and tactile examinations are the most common methods used in caries lesions assessment in clinical practice [1, 2]. Despite having been extensively used for detecting caries lesions, tactile exams have been advocated since they permit to identify important features to consider during the clinical decision-making process. Tactile examination allows the clinician to assess the surface texture of enamel and dentine lesions and evaluate discontinuities or microcavitations of detected lesions [3–5].

Sharp dental explorers have been pointed out as inappropriate tools for assessing dental lesions [1, 6, 7] because they can irreversibly damage enamel [8–11]. Despite this fact, many general dentists still use these tools for tactile examinations [12]. The use of ball-ended probes has recently been recommended as an improved method for caries assessment [3, 13, 14]. Although using sharp explorers can better distinguish between standards of different roughness [15], the ball-ended probe seems to be probably safer because it lacks a sharp extremity. However, no study has evaluated the effect of ball-ended probing on dental surfaces. Thus, we aimed to compare probing defects caused by ball-ended probes with sharp explorers on smooth and occlusal surfaces of primary teeth. The current exploratory study pioneered the use of environmental scanning electron microscopy (ESEM) to assess damage to the dental surface. This is a non-destructive technology that permits the longitudinal evaluation of dental damage.

## Methods

### Design

This study was approved by the Ethical Research Committee of the Dental School, University of Sao Paulo, Brazil (Protocol 181/2009). Primary teeth were donated by children from dental clinics of Department of Pediatric Dentistry, University of Sao Paulo, Brazil. All children who had teeth extracted or exfoliated during the sample collection period (2010–2011) were invited to participate, as long as the tooth had been in the oral cavity for at least 2 years. Each child’s parents or guardians provided consent for the tooth donation. Both sound teeth and those presenting caries lesions were included. Teeth with developmental defects or damaged during extraction were excluded. Teeth were stored in saline solution for up to 1 month. They were maintained in the solution until the end of ESEM captures.

**Fig. 1:**
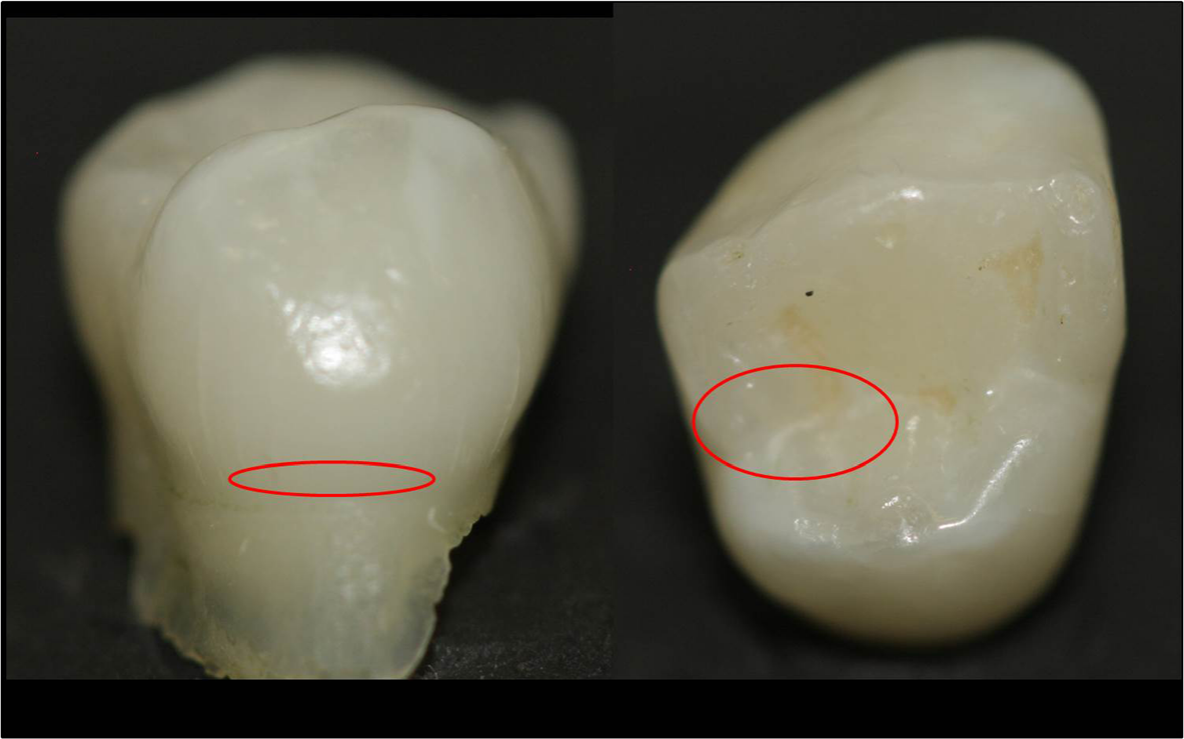
Schematic representation of area selected for ESEM capture

An external operator (undergraduate student) took pictures of tooth surfaces and defined an area within each obtained image. Selected areas were equivalent to plaque stagnation areas on smooth or occlusal surfaces (Fig. 1). These pictures were used as reference for ESEM captures.

One of the researchers (Associate Professor in Pediatric Dentistry and experienced in carried out studies in caries detection) visually classified the surfaces according to their type (smooth or occlusal) and baseline visual condition. These surfaces were positioned about 30 cm from examiner’s eye and were examined with the aid of a light reflector and air drying. No probe was used in this stage. Surface condition was classified according to the merged codes of International Caries Detection and Assessment System (ICDAS) (https://www.icdas.org/what-is-icdas): sound surfaces (without a change in enamel translucency after 5 s of air drying); initial or moderate caries lesions (surfaces with opacity, presenting or not visible surface discontinuity on enamel) or extensive caries lesions (cavities exposing dentine) [16].

Forty-nine surfaces of primary molars were randomly assigned to a tactile examination group according to probe type (ball-ended probe or sharp explorer - Golgran, São Paulo, Brazil). The allocation was done using Medcalc software (version 12.7.1.0, Mariakerke, Belgium). Two graduate students in Pediatric Dentistry, who used to participate in clinical studies focused on caries detection/management, probed the selected surfaces. They were orientated by the previously mentioned professor to assess the selected surfaces in order to detect characteristics of activity status of caries lesions [17]. As one of these characteristics was the texture, the researcher indicated that one or other instrument should be gently used for such purpose [18, 19]. During the examinations, one of the instruments was provided to the examiners. Each instrument was used for only five assessments to minimize the effect of tip wear. To guarantee that examiners were unaware of the aim of the study, other variations during examinations were proposed but changing the sample. This methodological strategy was only used to avoid interferences of the examiner favoring one of the probes and obviously, data related to other sources of variation were not included in this manuscript.

### Ultrastructural damage assessment

The ultrastructural damage was set as the outcome for this study. Images of the specimens were captured using an ESEM (ESEM 2020 Electroscan, Philips, Eindhoven, The Netherlands) at × 140–× 150 magnification to ensure that no surface alterations or defects would be missed. When necessary, morphological evaluations of occlusal surfaces were performed at × 1000 magnification. All defined areas from surfaces were evaluated using this process before and after probing. Teeth were re-hydrated after all ESEM captures.

**Fig. 2:**
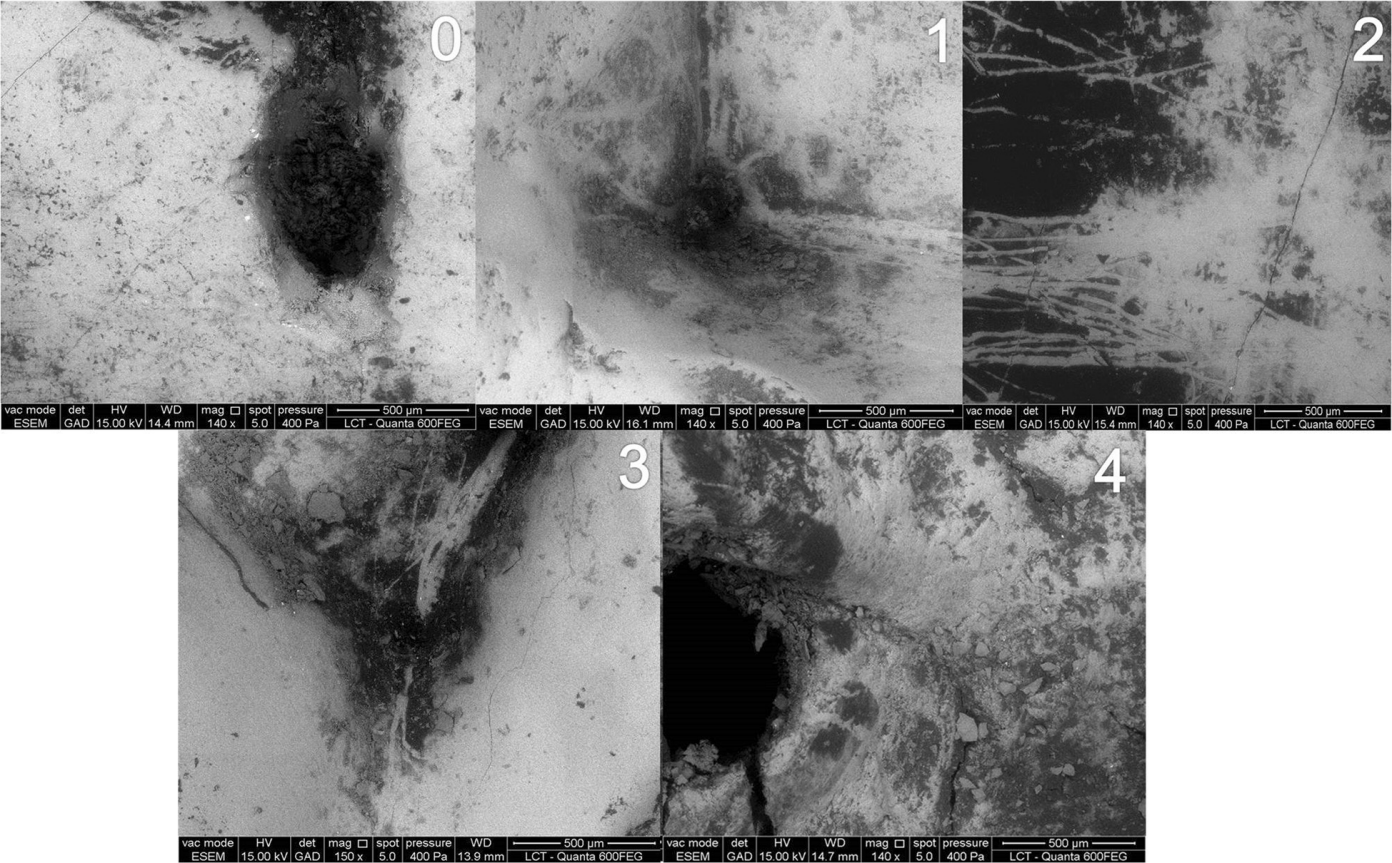
Criteria for classifying surfaces ultrastructural damage after probing – adapted from Kuhnisch et al., [9]

Two trained senior lecturers, experienced in evaluating dental SEM, were previously calibrated and performed pairwise evaluations of ESEM images (before vs. after probing). Examiners were blinded to the type of probing each tooth had received. They classified surface damage observed in the final image taking into account the surface condition in the initial image. They scored each pair according to an adaptation of a published criteria [9]: 0) no damage, 1) slight probe marking without defects, 2) distinct probe marking without defects, 3) distinct probe marking with discontinuity, and 4) enamel break-offs (Fig. 2). After 1 month, they also assessed individual images without knowing whether it was captured before or after probing to check the validity of the pairwise evaluation (i.e., to avoid overestimating damage in the after-probing set of images).

For both approaches, each examiner assessed the images independently. In case of disagreement, a consensus was reached in a joint session. They always evaluated images on the same computer screen in the same room. Finally, the examiners performed both pairwise and individual assessments on a subset of images (50 %), 1 month later, to verify intra-examiner reproducibility.

### Statistical analyses

Intra and interexaminer reproducibility were calculated using the weighted quadratic Kappa test. Multilevel Poisson regression analyses were performed to test associations between probe-induced damage and the surface type, surface condition, probe type and examiner. Only variables associated with a *p*-value ≤ 0.20 in unadjusted analyses were considered for entry into the model. Variables associated with a *p*-value ≤ 0.05 after adjustments were retained in the final models. The Wald test was used to derive *p*-values. Prevalence ratios or rate ratios with 95 % confidence intervals were calculated for each condition tested. The prevalence ratio was used when the outcome was dichotomized, i.e., when we considered presence vs. absence of damage to evaluated surfaces (models 1 and 2). Model 1 was based on pairwise evaluations. In model 2, outcomes were based on the assessment of individual images. A surface was considered damaged if the final score was greater than the initial one (based on individual evaluations). When we considered all possible final scores (0–4) as the outcome, the rate ratio was used to assess associations between damage and explanatory variables (model 3). In model 3, the initial score was used to adjust the final model.

All analyses were performed using the software MLwiN (version 2.10, Centre for Multilevel Modeling, Bristol, UK).

## Results

According to randomization process, the first examiner probed 13 surfaces using the sharp explorer and 13 different surfaces using the ball-ended probe. The second examiner probed 13 surfaces using the sharp explorer and 10 different surfaces using the ball-ended probe. Intra and interexaminer reproducibility for pairwise assessments were 0.99 and 0.88, respectively. For individual assessments these figures were 0.90 and 0.87, respectively.

The final sample consisted of 34 smooth surfaces and 15 occlusal surfaces. When grouped according to condition, the sample contained 12 sound surfaces, 29 surfaces with initial or moderate caries lesions, and eight surfaces with extensive caries lesions.

Images taken before probing revealed superficial damage to 63 % of the evaluated surfaces. Most of this baseline damage was classified as slight marks (61 %). Because there was no difference between the examiners, we analyzed their assessments together. Experimental probing caused additional damage to half of the examined surfaces (51 %). The most common defect was probe marks without discontinuity (72 %), which included slight marks (36 %) and distinct marks (36 %). Only 16 % of the surfaces had probe marks with discontinuity and 12 % had enamel break-offs.

When images were assessed in pairs, surfaces examined using the ball-ended probe had 72 % fewer defects than surfaces probed with the sharp explorer. The examiner and the surface condition were not associated with damage to these surfaces (*p* > 0.05) (Table 1, model 1). Although using the ball-ended probe caused some surface damage (22 %), no discontinuity or enamel break-off was caused by this type of probing.

**Table 1:** Multilevel analysis of association between probe-induced ultrastructural damage and exploratory variables – Model 1: pairwise assessment of ESEM images (outcomes: 0-no damage vs. 1-damage; Model 2: transition of scores given on individual assessment of ESEM images (0-no damage vs. 1 –damage); Model 3: individual assessment of final scores (outcome: scores 0 to 4) adjusted for baseline scores)

	Model 1				Model 2		Model 3			
Independent variables	n (%) defects after probing^a^without with		Prevalence^b^ ratio (95 % CI)	*p* value	Prevalence^b^ ratio (95 % CI)	*p* value	Rate ratio^b^(95 % CI)	*p* value	Rate ratio^c^(95 % CI)	*p* value
Surface type	17 (34.70)			0.87		0.55		**0.001**	-	-
Smooth (ref.)		17 (34.70)								
Occlusal	8 (16.32)	7 (14.28)	1.07(0.46 to 2.47)		1.36 (0.49 to 3.74)		2.06 (1.33 to 3.17)			
Surface condition	6 (12.24)	7 (14.28)		0.59	0.67 (0.24 to 1.89)	0.50	1.23 (0.70 to 2.18)	**0.001**	-	-
Sound (ref.)										
					0.31 (0.04 to 2.57)		2.55 (1.34 to 4.86)			
Initial or moderate Extensive	17 (34.70)	12 (24.48)	1.27 (0.50 to 3.22)							
	2 (4.08)	5 (10.20)	0.62 (0.13–3.06)							
Probe type	5 (10.20)	18 (36.73)		**0.01**	0.38 (0.12 to 1.17)	0.09	0.81 (0.52 to 1.28)	0.74	0.38 (0.12 to 1.17)	0.09
Ball-ended (ref.)										
Sharp explorer	20 (40.81)	6 (12.24)	0.28 (0.11–0.76)							
Examiner	13 (26.53)	13 (26.53)		0.91	1.88 (0.68 to 5.18)	0.28	0.98 (0.63 to 2.51)	0.91		
First (ref.)										
Second	12 (24.48)	11 (22.44)	1.04 (0.48–2.29)							

-Variable was tested, but not associated with the outcome in the multiple model

Figures in bold symbolize statistically significant differences in each unadjusted model

^a^Number of defects based on pairwise evaluation of ESEM images

^b^Unadjusted analysis. No multiple model was performed because only one variable was selected to enter into multiple models (*p* < 0.20)

^c^Adjusted analysis (baseline score used for adjustment)

Surface damage was not associated with the type of probe when models 2 and 3 were performed (Table 1).

**Fig. 3:**
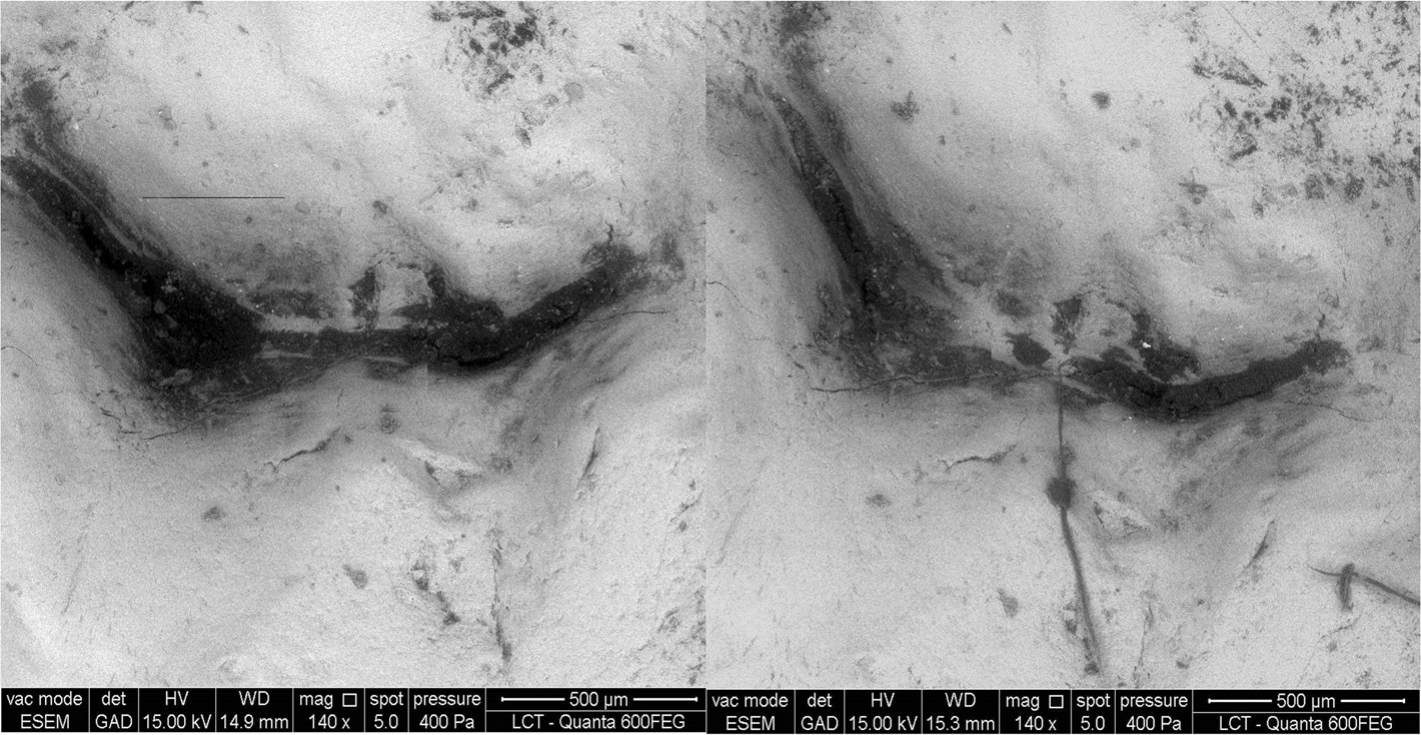
Images before and after probing. Defects can be observed in the initial and final images. Note that they could have received the same score in the individual analysis, but in the pairwise comparison, we could notice probing effect was worsen

For some pairs of ESEM images, pairwise evaluation identified probe damage, whereas individual assessments did not (Fig. 3).

## Discussion

Our findings showed that the use of ball-ended probes causes less ultrastructural damage to dental surfaces than the use of sharp explorers. In fact, probing with a sharp explorer caused some type of damage to most examined surfaces, as observed in previous reports [8-11, 20]. Although the use of non-sharp explorers or ball-ended probes has been recommended [3, 13, 14], damage caused by these types of probes has not been investigated. This study is the first to explore that ball-ended probes may cause less damage than sharp explorers when performing caries diagnostic procedures.

This study was also the first to use ESEM to assess damage resulting from probing. Because ESEM does not require specimen metallization, it is possible to obtain images from the same tooth region before and after probing. This reduces the number of confounding factors and improves the accuracy of damage evaluation. Traumatic effects caused by probing have been typically evaluated using light microscopy of tooth sections [8, 11] and destructive methodologies as scanning electron microscopy [9]. Using these methods, investigators must use different samples or different parts of the same lesion to perform their analysis, i.e., longitudinal assessments are not possible. As such, intrinsic and local variations between the compared samples may affect the results.

The pairwise evaluation of ESEM images identified changes caused by probing, even if baseline marks were present in the surfaces and probing could have worsen this condition. This type of changes was not usually apparent when images were analyzed individually. As we used teeth exposed to the oral environment, probably, most teeth had been previously probed. Visible marks in the baseline images likely resulted from these previous probing procedures, and partially compromised our evaluations. Slight differences between the pairwise and individual analyses had been expected, as individual evaluations do not take into account the initial conditions of the teeth. We conducted both individual and pairwise evaluations to minimize potential overestimation of damage in the final images. However, model 1 (based on pairwise analysis) represented the results of this study more accurately and yielded higher discriminatory power regarding the effects of sharp explorers and ball-ended probes. The individual assessments validated the pairwise comparison and verified that they were not biased.

The observed ultrastructural damage caused by ball-ended probes seems to be less severe than those caused by sharp explorers, probably due to differences in probes. Even when using ball-ended probes, therefore, the need to probe gently should be stressed to minimize the harmful effects of probing. Explorers could therefore generate cavitations within continuous demineralized areas of the enamel, accelerating lesion progression [8-11].

A previous study has demonstrated that sharp explorers can more effectively distinguish differences in surface roughness than a ball-ended probe [21]. Indeed, the accurate evaluation of surface roughness is important to detect caries lesions correctly. Despite different recommendations concerning the use of sharp explorers or ball-ended probes around the world [13], it should be questioned if a tool that can better identify some characteristics of a caries lesion, but increases the risk of damaging the lesion. In other words, an accurate evaluation should not sacrifice the integrity of the examined surface. Furthermore, it is still unclear if performance differences between sharp explorers and ball-ended probes in characterizing lesion roughness [15] are reflected in relevant clinical endpoints, such as caries-lesion progression and other patient-centered outcomes.

Previous studies have reported more extensive probing damage than we observed in the present protocol. In general, studies related to probing damage have been conducted using third molars, for which the length of exposure to the oral environment was not reported [8, 9]. These molars, therefore, could have had very different mineralization levels. This is important because recently erupted teeth tend to be more susceptible to damage. Even thinner and less mineralized than permanent teeth, evaluated primary teeth that had been in the oral cavity for more than 2 years, thereby minimizing the effect of post-eruptive maturation of enamel [21, 22].

Another concern about tactile examinations is the force with which the probe is used [13]. Force is a subjective action influenced by hand position, training, experience, fatigue, muscle strength, body weight of the dentist, and other factors [23]. In this way, the calibration of probing pressure could not be relevant to clinical practice [9]. On the other hand, when probing a surface to assess roughness or texture, it is important to use the probe gently. Therefore, in this study, we tried to standardize the probing force between examiners giving them preliminary instructions for probing gently for caries activity assessment and involving experienced examiners in caries detection in clinical trials.

Given the characteristics of this exploratory study, as the sample size and composition, we avoided making inferences in such cases in which no significant difference was observed. The statistical power may depend on the magnitude of the effect and the sample size. Therefore, we cannot assume that differences not evidenced for some variables are actually absence of differences or a result of losing power in some analyses [24]. On the other hand, even using a small sample size, the effect of probing could be observed, showing the effect is large enough to be demonstrated even in a small sample. Thus, we believe the findings of this exploratory study are important to be reported.

Although the effect of probing marks on a long-term analysis has not been directly assessed, it is likely that these types of microscopic defects contribute to bacterial adhesion [25]. We speculate that regular polishing and toothbrushing could progressively remove slight marks. Indeed, the high proportion of slight marks observed in the pre-probing sample may reflect this process. Another concern relates to successive probing in the oral cavity. Enamel marks may become worse if the surface is repeatedly and roughly probed during each clinical examination. In addition, it is important to consider the enamel could have more prone to scratches after successive ESEM captures and the magnitude of the effects could be superior to real life. Since all surfaces were exposed to the same protocol and same number of ESEM captures, we do not believe this limitation could have impacted on our findings regarding the probes.

Certainly, this study does not reflect all clinical conditions; however, it could isolate and consequently clarify some effects of ball-ended probing on tooth surfaces that had never been evaluated. Further clinical studies should be conducted to investigate the impact of slight probing-related damage. Based on the severe ultrastructural damage caused by sharp explorers, both for clinical practice or further studies, we could advise not using them neither for gently removing plaque from nor performing tactile examination of dental surfaces as part of activity assessment of caries lesions.

## Conclusion

Ball-ended probes cause less ultrastructural damage than sharp dental explorers. However, it is important to emphasize the importance of gentle probing even when using the ball-ended probe.

## Abbreviations

CI: confidence interval
ESEM: environmental scanning electron microscopy
PR: prevalence ratios
